# Microbiome analysis of bronchoalveolar lavage (BAL) specimens from immunocompromised patients with pneumonia compared to those from healthy volunteers

**DOI:** 10.1371/journal.pone.0351562

**Published:** 2026-06-10

**Authors:** Zareen Fatima, Matthew D. Surette, Sarah Marttala, Daniela Leto, Padman Jayaratne, Fiona Smaill, Marek Smieja, Mohammad Rubayet Hasan

**Affiliations:** 1 Department of Pathology and Molecular Medicine, McMaster University, Hamilton, Canada; 2 Department of Laboratory Medicine and Pathobiology, University of Toronto, Toronto, Canada; 3 Research Institute of St. Joe’s Hamilton, Hamilton, Canada; 4 Hamilton Regional Laboratory Medicine Program, Hamilton, Canada; University of South Carolina School of Medicine, UNITED STATES OF AMERICA

## Abstract

**Background:**

Metagenomic sequencing of bronchoalveolar lavage (BAL) specimens is increasingly being applied for the diagnosis of lower respiratory tract infections, offering agnostic pathogen detection and a faster turnaround time. While metagenomic sequencing of BAL specimens can reveal a wide range of organisms, their clinical relevance is often unclear because of the challenge of distinguishing true pathogens from background taxa. This study compared the BAL microbiomes of immunocompromised patients with pneumonia to those of healthy volunteers, with the aim of assisting clinical interpretation of metagenomics-based approaches for diagnosing pneumonia in this patient population.

**Methods:**

BAL specimens from healthy control volunteers (n = 20) were collected during a COVID-19 vaccine trial, while residual BAL specimens from immunocompromised patients (n = 52) were obtained from the Hamilton Regional Laboratory Medicine Program (HRLMP) after standard culture and PCR testing. 16S rRNA gene amplicon sequencing was performed using Nanopore technology. Reads were classified using Minimap2 in EPI2ME, and microbiome analyses were conducted using the *vegan* and *MaAsLin2* packages in RStudio (v2026.1.1.403).

**Results:**

Immunocompromised patients showed significantly lower bacterial read counts and reduced alpha diversity (p < 0.0001; Wilcoxon Rank-Sum test), along with higher inter-sample heterogeneity. In contrast, BAL samples from healthy controls exhibited a more homogeneous microbial profile dominated by anaerobic Gram-negative genera, including *Prevotella, Veillonella, Selenomonas*, and *Fusobacterium*. Beta diversity analyses using Bray–Curtis and Jaccard distance metrics demonstrated significant compositional separation between cohorts (PERMANOVA p = 0.001), with tight clustering of healthy controls and marked dispersion among immunocompromised samples. Differential abundance analysis identified 96 significantly altered species (q < 0.05), with immunocompromised patients showing depletion of anaerobic commensals and enrichment of clinically relevant pathogens, including *Stenotrophomonas maltophilia*, *Enterococcus* spp., *Mycoplasma* spp., and *Nocardia* spp.

**Conclusion:**

Immunocompromised patients demonstrated a markedly disrupted and heterogeneous BAL microbiome, characterized by a loss of anaerobic commensals and an enrichment of potentially pathogenic taxa. This study provides a characterization of the dysbiotic state in immunocompromised pneumonia, offering a baseline reference for future longitudinal studies and clinical trials aimed at improving the interpretation of metagenomic findings in this patient population.

## Introduction

The incidence of critically ill patients with compromised immune systems has surged, constituting roughly one-third of ICU admissions. This rise is attributed to aggressive and prolonged cancer treatments, increased transplantation, and the introduction of immune-modulating agents for autoimmune disorders. Consequently, many now live with heightened vulnerability to infections. Severe respiratory infections, such as pneumonia, are the primary cause for ICU admissions among these patients, often leading to acute respiratory failure and sepsis. Pulmonary infections are frequent in these patients, posing risks from both common and opportunistic bacterial or fungal infections [1,2].

Bronchoalveolar washing or bronchoalveolar lavage (BAL) is commonly used for investigating the etiology of lung infiltrates in immunocompromised patients, with reported diagnostic yields varying from 26% to 69% [3]. Microbiological workups include routine microscopy and culture, and PCR for selected pathogens. However, the diagnostic yield of these tests is significantly impacted by early, empiric antibiotic therapy. Furthermore, many atypical pathogens, slowly growing organisms, or unculturable pathogens may be missed by standard methods. Establishment of a next-generation sequencing (NGS)-based, 16s rRNA gene sequencing approach to detect a wide range of bacterial pathogens in BAL specimens may improve the microbial diagnosis of pneumonia in immunocompromised patients. However, knowledge of commensal flora is critical for developing a metagenomics-based diagnostic tool to detect pathogens in BAL.

Microbiome analysis of bronchoalveolar lavage (BAL) fluid has emerged as a cornerstone in exploring the microbial ecology of the lower respiratory tract, offering critical insights into health and disease through culture-independent techniques. BAL sampling typically involves instilling sterile saline into a targeted airway segment via bronchoscopy, followed by retrieval of fluid that reflects the epithelial lining fluid microbiota and potential pathogens [4]. Using 16S rRNA gene amplicon sequencing and metagenomic next-generation sequencing (mNGS), researchers have identified core bacterial communities across pediatric and adult cohorts in a limited number of studies. For instance, a study analyzing pediatric BAL specimens identified 21 unique taxa—including genera such as *Streptococcus*, *Granulicatella*, *Prevotella*, and *Veillonella*—present in at least 30–50% of samples from non-immunocompromised children [5]. Similarly, in healthy young adults, the lower airway microbiota (from bronchial brushing and bronchoalveolar lavage) was found to be dominated by *Prevotella* and *Veillonella* species [6]. Microbiome analysis of BAL has also been conducted in various lung conditions, such as lung cancer, bronchiectasis, chronic obstructive pulmonary disease (COPD), pulmonary fibrosis, and *Mycoplasma pneumoniae* infection, revealing significant microbial imbalances and associations with disease severity [7–10]. However, data on the lower respiratory tract microbiota in immunocompromised patients remain limited.

In this study, we compared the microbiomes of BAL specimens collected from immunocompromised patients with pneumonia, with those from healthy individuals using 16S rRNA gene sequencing with Nanopore technology.

## Materials and methods

### Study design

This retrospective observational study was conducted at the Research Institute of St. Joe’s, Hamilton, ON, using residual specimens obtained from the Hamilton Regional Laboratory Medicine Program (HRLMP) Microbiology Laboratory. Specimens were collected after standard culture and PCR testing (S1 Table) for the immunocompromised cohort, and from a COVID-19 vaccine trial for the healthy cohort. All clinical specimens were anonymized prior to use in the study. Standard microbiological test results, radiological findings (whenever applicable) and clinical data, including comorbidities and the use of immunosuppressive and antimicrobial drugs, were collected through retrospective chart review on July 14, 2025, and March 10 – April 10, 2026. The study was approved by the Hamilton Integrated Research Ethics Board (HiREB) (Protocol numbers # HiREB 14230 [11] and #18181).

### Sample collection and processing

For the immunocompromised cohort, between October 2022 and January 2025, residual BAL specimens (n = 61) were stored at –80 °C for microbiome analysis following PCR testing for *Pneumocystis jirovecii*. Specimens were selected based on *P. jirovecii* PCR testing, as this test is exclusively performed on immunocompromised patients at HRLMP; specimens from any other patient population submitted for *P. jirovecii* PCR are rejected by the testing laboratory. Specimens with insufficient volumes for analysis were excluded. A total of eight specimens were excluded from the analysis due to duplicate samples from the same patients. These samples were typically collected from different lung sites (e.g., right upper or middle lobe vs. right lower lobe). For duplicate samples, specimens were selected from the affected areas of the lungs based on radiological findings (chest X-ray or CT). Prior to *P. jirovecii* PCR testing, these specimens had already undergone standard bacterial and fungal cultures, as well as PCR testing for selected bacterial, viral, and fungal pathogens. While bacterial culture on majority of these specimens resulted in no growth or insignificant growth (low colony count or growth of normal flora), a few samples were positive for bacterial, fungal or viral pathogens (S1 Table).

For the healthy control cohort, BAL specimens (n = 20) were obtained from a Phase I open-label clinical trial evaluating the safety and immunogenicity of ChAd68 and AdHu5 vector-based trivalent COVID-19 vaccines delivered via inhaled aerosol. Detailed inclusion and exclusion criteria for the recruitment of study participants have been described previously [11]. Briefly, healthy adults aged 18–65 years were eligible for inclusion. Participants had no history of COVID-19 infection or a documented infection at least six months prior. Key exclusion criteria included pregnancy or lactation, significant acute or chronic illness or immunosuppressive therapy, bleeding disorders, chronic respiratory diseases requiring regular treatment, current or recent smoking (within one year), abnormal baseline spirometry, contraindications to bronchoscopy, clinically significant abnormal laboratory results, and substance use that could interfere with study participation. BAL specimens from healthy controls were collected between February 2022 and February 2024, then aliquoted and stored at –80 °C. All specimen processing was conducted in a biosafety level 2 laboratory in accordance with standard precautions to prevent contamination.

### 16s rRNA gene sequencing and analysis

The 16S rRNA gene (~1.4 kb) was amplified using forward primer 16S-8F (AGAGTTTGATCATGGCTCAG) and reverse primer 1392R (ACGGGCGGTGTGTRC), as described previously [12,13]. The 16S rRNA gene amplification and sequencing workflows were first verified using a panel of 15 cultured bacterial species, including ATCC reference strains and culture-confirmed clinical isolates (S2 Table and S1 Fig). 16S sequencing correctly identified all isolates at the species level except for *Escherichia coli*, for which closely related *Escherichia* species were detected that are known to be indistinguishable from *E. coli* by 16S rRNA gene sequencing. Total nucleic acids from BAL specimens were extracted using the EasyMag platform (bioMérieux) according to the standard protocol. For each specimen, 5 µL of eluate was added to a master mix consisting of 0.2 µM of each primer, 1 × Q5 High-Fidelity Master Mix, 0.8 × EvaGreen, and nuclease-free water to a final volume of 25 µL. PCR was performed with an initial denaturation at 98 °C for 2 minutes, followed by 40 cycles of denaturation at 98 °C for 10 seconds, annealing at 55 °C for 30 seconds, and extension at 72 °C for 1 minute. A final extension was carried out at 72 °C for 5 minutes. Five microliters of PCR product was used to prepare sequencing libraries using the Native Barcoding Kit 96 V14 (Oxford Nanopore), and sequencing was performed on MinION R10.4.1 flow cells using a GridION device for 24 hours according to the manufacturer’s instructions. Super-accurate basecalling, quality filtering (minimum quality score = 10), and demultiplexing were performed using MinKNOW software. Each sequencing batch included a positive control containing a known bacterial species and a blank control consisting of nuclease-free water.

### Microbiome profiling

Basecalled and demultiplexed FASTQ files generated from Nanopore sequencing were processed using the *wf-16s* workflow within the EPI2ME suite (https://epi2me.nanoporetech.com/epi2me-docs/workflows/wf-16s/). Reads were subjected to quality control filtering, retaining sequences with a minimum Q score of 15 and lengths between 1300 and 1700 bp to capture near full-length 16S rRNA gene amplicons. Following filtering, reads were aligned using minimap2 against the ncbi_16s_18s_28s_ITS reference database (https://www.ncbi.nlm.nih.gov/refseq/targetedloci/) for taxonomic classification. Low-quality and unclassified reads were excluded from downstream analyses. Taxonomic assignments were generated at multiple levels (phylum to species, where possible), and an abundance table was produced summarizing read counts per taxon across all samples.

Downstream microbiome analyses were performed in RStudio (R version 2026.1.1.403) using the abundance tables generated from the EPI2ME wf-16s workflow. Analyses were conducted using R packages including tidyverse [14], vegan [15], ape [16], ggplot2 [17], and Maaslin2 [18](https://github.com/mrubayethasan/Microbiome-Analysis). Raw count data were imported and organized into a taxa-by-sample matrix, with samples matched to corresponding metadata. Taxonomic strings were parsed to extract hierarchical levels (phylum to species). Relative abundances were calculated by normalizing counts to the total sequencing depth per sample. Community composition was summarized at the phylum, genus, and species levels. For visualization, the top 10 most abundant taxa at each level were selected based on total relative abundance across samples, with remaining taxa grouped as “Other.” Stacked bar plots were generated to display taxonomic profiles across samples and study groups. For heatmap visualization, the top 20 species based on total abundance were selected, log-transformed, and hierarchically clustered using Euclidean distance.

Alpha diversity metrics, including observed richness, Shannon index, Simpson index, and Chao1 estimator, were calculated using the vegan package. Group comparisons were performed using the Wilcoxon rank-sum test. Beta diversity was assessed using Bray–Curtis dissimilarity (abundance-based) and Jaccard distance (presence/absence-based). Principal coordinates analysis (PCoA) was performed using the ape package, and group differences were evaluated using permutational multivariate analysis of variance (PERMANOVA) implemented in the vegan package (adonis2 function). For differential abundance analysis, taxa were aggregated at the species level, and low-abundance features were filtered based on total read counts. Relative abundance data were analyzed using the Microbiome Multivariable Associations with Linear Models (Maaslin2) package with group as a fixed effect with log transformation. Statistical significance was determined using false discovery rate (FDR)-adjusted q-values, with a threshold of q < 0.1. Results were visualized using forest plots displaying effect sizes and corresponding confidence intervals. All plots were generated using ggplot2 and related visualization packages.

## Results

### Study population

The microbial composition of bronchoalveolar lavage (BAL) specimens collected from 52 immunocompromised patients were compared with 20 BAL specimens from 20 healthy control volunteers enrolled in a COVID-19 vaccine trial. Immunocompromised patients were older than healthy controls and included a lower proportion of females (Table 1). This cohort was characterized by a high burden of underlying conditions, most commonly malignancy (51.9%), along with smaller proportions of patients with HIV infection, chronic obstructive pulmonary disease, and other comorbidities. Many patients were receiving therapies (80.8%) associated with immunosuppression, including chemotherapy or targeted treatments, immunosuppressive medications, and, in some cases, solid organ or hematopoietic stem cell transplantation. Notably, the majority (71.2%) had been exposed to antibiotics prior to bronchoscopy. The remaining patients also likely received antibiotics as part of standard clinical practice; however, this information was not documented in the available patient charts. The antibiotic regimens were heterogeneous and included broad-spectrum antibacterial agents (e.g., ceftazidime, ceftriaxone, meropenem, piperacillin–tazobactam, levofloxacin, and vancomycin), as well as trimethoprim–sulfamethoxazole and, in some cases, anti-viral and antifungal agents such as acyclovir, fluconazole, caspofungin, and voriconazole.

**Table 1:** Baseline characteristics of healthy controls and immunocompromised patients included in BAL microbiome analysis.

Characteristic	Healthy Cohort (n = 20)	Immunocompromised Cohort (n = 52)
**Age, mean ± SD (years)**	37.9 ± 14.3	58.0 ± 16.8
**Female sex, n (%)**	12 (60.0%)	20 (38.5%)
**Comorbidities, n (%)**		
Cancer	–	27 (51.9%)
HIV	–	3 (5.8%)
COPD	–	3 (5.8%)
Other	–	12 (23.1%)
**Treatment, n (%)**		
Transplant recipient	–	7 (13.5%)
Chemotherapy/targeted therapy	–	17 (32.7%)
Immunosuppressive therapy	–	29 (55.8%)
**Received antibiotics prior to bronchoscopy, n (%)**	–	37 (71.2%)

### Microbiome profiles

For microbiome profiling of BAL samples, the nearly full-length 16S rRNA gene covering hypervariable regions V1 to V8 was amplified (12, 13) and sequenced using Nanopore sequencing, which enables long-read sequencing and provides uniform coverage across the 16S rRNA gene. A total of 7.83 million base-called sequencing reads (4.1 Gb, N50 = 647 bp) were obtained from 20 specimens in the healthy control cohort, compared to a total of 14.5 million reads (7.94 Gb, N50 = 608 bp)(from two sequencing runs) from 52 specimens in the immunocompromised cohort. Although most reads were shorter than the expected amplicon size (~1.4 kb), likely due to host DNA contamination, a distinct peak corresponding to 16S rRNA gene amplicons within the expected size range was observed in all sequencing batches (S2 Fig). Raw sequencing data were therefore processed in EPI2ME to exclude reads outside the 1300–1700 bp range. Additionally, to minimize taxonomic misassignments resulting from base-calling errors in Nanopore sequencing, we applied a strict Q score threshold of 15, corresponding to 95% base accuracy.

**Fig 1:**
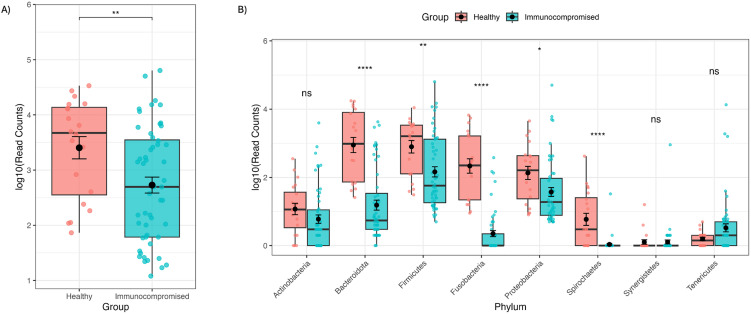
16S rRNA gene metagenomic sequencing output from BAL specimens from healthy control volunteers and immunocompromised patients with pneumonia. **A)** Comparison of average bacterial read counts between healthy control and immunocompromised cohorts. **B)** Comparison of average bacterial read counts by phylum between healthy control and immunocompromised cohorts. *p*-values were obtained using the Wilcoxon Rank-Sum test.

The mean number of bacterial reads obtained from the healthy control cohort was significantly higher (~1.8 fold) than that of the immunocompromised cohort (Wilcoxon Rank-Sum Test, *p* < 0.01) (Fig 1A). Based on absolute total read counts, the most abundant taxa at the phylum level in the healthy control cohort were *Bacteroidota* (90,255), *Firmicutes* (45,223) and *Fusobacteriota* (23,686). In contrast, the most abundant phyla in the immunocompromised cohort were *Firmicutes* (137,403), *Proteobacteria* (70,186) and *Tenericutes* (17,091). There was no significant difference in mean bacterial reads between the healthy control and immunocompromised cohorts for the phyla *Actinobacteria*, *Synergistetes* and *Tenericutes* (Wilcoxon Rank-Sum Test; Fig 1B). However, the mean bacterial reads in the healthy control cohort were significantly higher than those in the immunocompromised cohort for the phyla *Bacteroidota* (*p* < 0.0001), *Firmicutes* (*p* < 0.01), *Fusobacteriota* (*p* < 0.0001), *Proteobacteria* (*p* < 0.05) and *Spirochaetes* (*p* < 0.0001).

### Microbial diversity

**Fig 2:**
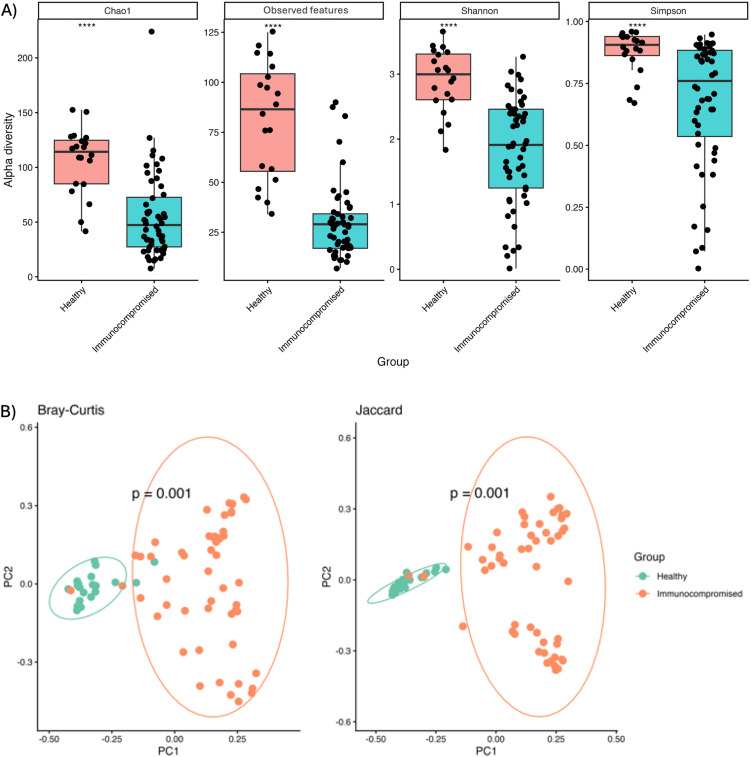
Comparison of microbial diversity between BAL specimens from healthy control volunteers and immunocompromised patients with pneumonia. **A)** Alpha diversity, calculated using Observed features, Chao1, Shannon, and Simpson indices. **B)** Beta diversity, calculated using Bray–Curtis and Jaccard dissimilarity indices. For alpha diversity *p*-values were obtained using the Wilcoxon Rank-Sum test; for beta diversity *p*-values were obtained from PERMANOVA for both Bray-Curtis and Jaccard dissimilarity indices.

The diversity of microbial communities in BAL specimens from the healthy control and immunocompromised cohorts was compared using various alpha and beta diversity indices. Alpha diversity metrics—including Observed features, Chao1 (which estimates species richness), Shannon index (which estimates species diversity) and Simpson index (which accounts for both species richness and relative abundance)—were all significantly higher (*p* < 0.0001) in the healthy control cohort compared to the immunocompromised cohort (Fig 2A). The ordination analyses based on Bray–Curtis and Jaccard dissimilarities both revealed clear separation of microbial community composition between the two study groups (Fig 2B). In the Bray–Curtis PCoA, samples from immunocompromised individuals formed a broad, dispersed cluster, indicating greater inter-individual variability in community structure. In contrast, healthy samples clustered tightly, suggesting a more homogeneous microbial composition. The separation between groups was statistically significant (PERMANOVA, *p* = 0.001), supporting distinct differences in relative abundance–based community structure. Similarly, the Jaccard PCoA, which reflects differences in community membership (presence/absence), showed a pronounced separation between healthy and immunocompromised samples (PERMANOVA, *p* = 0.001). As with Bray–Curtis, the immunocompromised group exhibited greater dispersion, while healthy samples remained tightly grouped. This indicates that both the composition and the membership of microbial communities differ significantly between groups.

### Relative abundance

**Fig 3:**
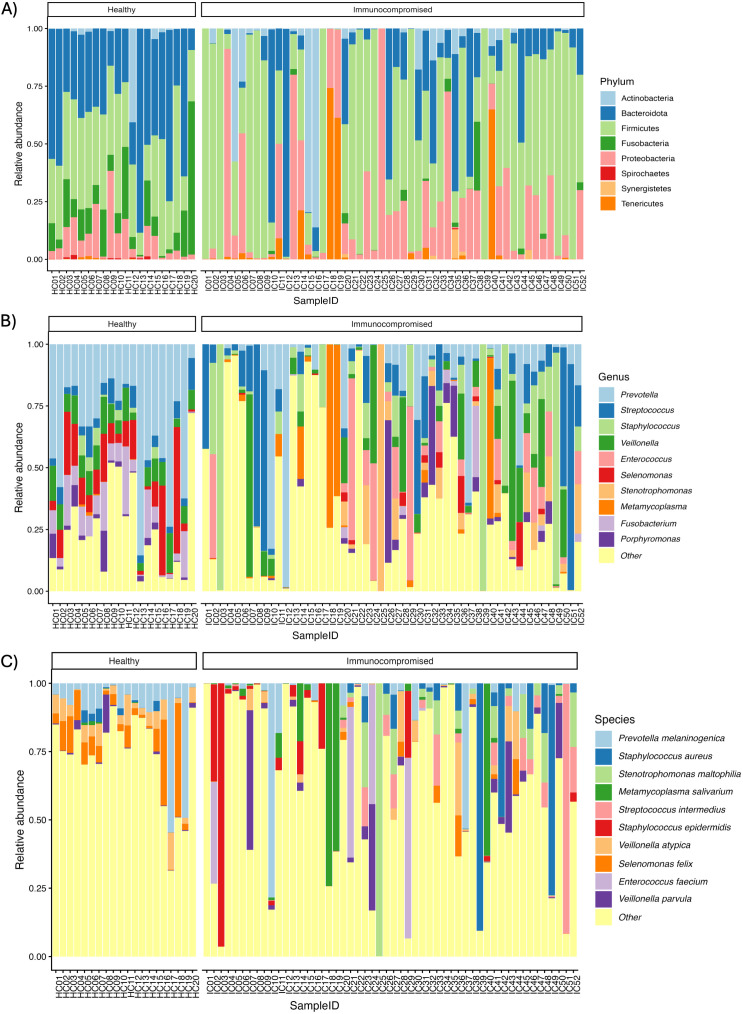
Comparison of relative abundance of bacterial taxa between BAL specimens from healthy control volunteers and immunocompromised patients with pneumonia. **A)** Relative abundance at the phylum level. **B)** Relative abundance at the genus level. **C)** Relative abundance at the species level.

We compared the relative abundance of taxa at the phylum, genus, and species levels (Fig 3). At the genus and species levels, only the top 10 most abundant taxa are shown, with all remaining taxa grouped into an “Other” category. At the phylum level (Fig 3A), microbial communities in healthy controls were dominated by *Bacteroidota* and *Firmicutes*, with consistent contributions from *Fusobacteriota* and relatively low abundance of other phyla. In the immunocompromised cohort, *Firmicutes* remained a dominant phylum in most samples, with occasional increases in *Actinobacteria*, *Proteobacteria*, and *Tenericutes*. At the genus level (Fig 3B), healthy samples displayed relatively consistent community structures, largely dominated by *Prevotella*, *Veillonella, Selenomonas* and *Fusobacteria*. In contrast, the immunocompromised cohort showed increased variability in genus-level composition, with frequent prominence of genera such as *Stenotrophomonas*, *Enterococcus*, *Streptococcus* and *Staphylococcus*. The relative contribution of the “Other” category was also higher and more variable in immunocompromised samples, reflecting a more uneven distribution of taxa. At the species level (Fig 3C), healthy controls were characterized by consistent presence of species such as *Prevotella melaninogenica*, *Veillonella atypica* and *Selenomonas felix*. In contrast, immunocompromised samples demonstrated marked inter-individual variability, with intermittent dominance of species such as *Stenotrophomonas maltophilia*, *Enterococcus faecium*, and *Staphylococcus epidermidis*. The increased proportion of the “Other” category in several immunocompromised samples further highlights the greater diversity and compositional heterogeneity within this group.

### Clinically significant taxa

**Fig 4:**
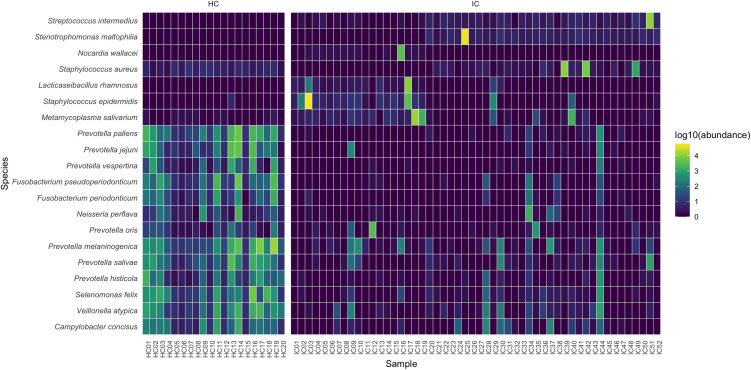
Heatmap of species-level abundance across BAL specimens. Heatmap showing the absolute abundance (log10-transformed) of the top 20 species ranked by total abundance across all bronchoalveolar lavage (BAL) samples from healthy controls and immunocompromised cohorts. Species were selected based on overall abundance, log-transformed, and hierarchically clustered using Euclidean distance.

To visualize clinically relevant taxa in immunocompromised patients relative to healthy subjects at the individual sample level, we generated a heatmap of the absolute (log10-transformed) abundance of the top 20 most prevalent species across all samples (Fig 4). Clear differences in species-level composition were observed between the two cohorts. In the healthy cohort, microbial communities were consistently dominated by anaerobic Gram-negative bacteria, which were present at relatively high and uniform abundances across samples, indicating a stable and homogeneous community structure. In contrast, the immunocompromised cohort showed a marked reduction in these commensal species, with many samples exhibiting low or undetectable levels. Instead, this group displayed greater heterogeneity, with intermittent enrichment of opportunistic and potentially pathogenic species. Notably, *Stenotrophomonas maltophilia*, *Staphylococcus aureus*, *Staphylococcus epidermidis*, *Nocardia wallacei*, and *Streptococcus intermedius* were elevated in a subset of immunocompromised samples but were largely absent or present at low levels in healthy controls. Additionally, *Metamycoplasma salivarium* and *Lacticaseibacillus rhamnosus* showed variable but occasionally high abundance in immunocompromised samples. Among the immunocompromised cohort, four samples were culture-positive for clinically significant pathogens (one positive for *Stenotrophomonas maltophilia* and three for *Staphylococcus aureus*) (S1 Table; IC25, IC39, IC42, and IC45). In each case, 16S rRNA gene sequencing identified the same organism as the most abundant species, demonstrating concordance between culture-based and sequencing-based detection.

**Fig 5:**
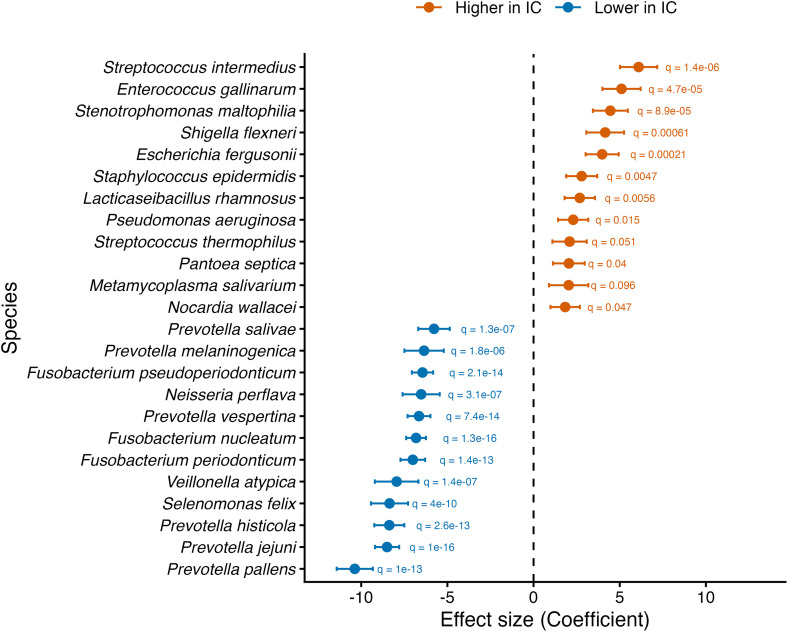
Differentially abundant species between healthy and immunocompromised cohorts identified by MaAsLin2. Forest plot showing the top differentially abundant species ranked by effect size (coefficient) from MaAsLin2 analysis comparing immunocompromised and healthy control cohorts. Points represent model coefficients (log fold change), and horizontal bars indicate the corresponding confidence intervals. Only species with *q*-values < 0.1 are shown, with *q*-values indicated alongside each feature.

Differential abundance analysis using MaAsLin2 further confirmed significant shifts in species composition between cohorts (S3 Table). A total of 104 species were differentially abundant (*q* < 0.1), of which 96 remained significant at *q* < 0.05. Of these, 15 species showed a positive log fold change (LFC), while the majority exhibited negative LFC, indicating depletion in immunocompromised individuals. The forest plot (Fig 5) highlights the top species ranked by effect size. Species with negative LFC were predominantly anaerobic commensals, including *Prevotella*, *Fusobacterium*, *Veillonella*, and *Selenomonas*. In contrast, species with positive LFC included opportunistic and clinically relevant taxa, such as *Streptococcus intermedius*, *Enterococcus gallinarum*, *Escherichia* spp., *Stenotrophomonas maltophilia*, *Mycoplasma* spp., *Pseudomonas* spp., and *Nocardia* spp., some of which were not detected by culture.

## Discussion

The lower respiratory tract (LRT), once considered a sterile environment, is now recognized as a complex microbial niche that plays an important role in respiratory health and disease. Advances in next-generation sequencing (NGS) techniques have revealed that the LRT harbors a diverse but low-biomass bacterial community, shaped by microaspiration from the upper airways, mucociliary clearance, and host immune responses. Dominant genera in healthy individuals often include *Prevotella*, *Veillonella*, and *Streptococcus*, which are believed to originate primarily from the oral cavity [19]. Disruption of this microbial balance—referred to as dysbiosis—has been associated with various pulmonary conditions, including chronic obstructive pulmonary disease (COPD), asthma, and pneumonia [7–10]. In immunocompromised individuals, dysbiosis may facilitate colonization by opportunistic pathogens, contributing to disease progression and poor clinical outcomes. Importantly, differentiating between commensal colonization and true infection remains a clinical challenge, particularly in vulnerable populations. Therefore, in this study, we compared the microbiota of BAL specimens from immunocompromised patients with pneumonia to that of healthy controls, using Nanopore sequencing of bacterial 16S rRNA gene sequences.

The microbiome composition of BAL specimens collected from healthy controls in our study aligns well with previous reports in adults, showing a predominance of anaerobic Gram-negative bacterial species such as *Prevotella* sp., *Veillonella* sp., *Selenomonas* sp., and *Fusobacterium* sp. [20]. However, compared to the BAL microbiota of pediatric populations reported in earlier studies, the presence of *Streptococcus* sp. and *Granulicatella* sp. was less prominent in our healthy adult cohort.

In contrast to the healthy control cohort, the immunocompromised cohort exhibited significantly lower overall 16S rRNA gene read counts, although BAL specimens from patients with pneumonia would be expected to have a higher bacterial load due to pathogen overgrowth. However, it should be noted that a large proportion (>70%) of patients in this cohort had received broad-spectrum antimicrobial therapy prior to bronchoscopy, which is expected to reduce microbial burden and consequently diminish bacterial DNA yield. Such pre-treatment may explain the lower 16S rDNA read output observed despite the presence of clinical infection. Technical factors, such as PCR inhibition, DNA extraction efficiency, or the presence of host DNA, may also have contributed to these findings; however, this is less likely, as BAL specimens from both cohorts were collected and processed using the same standardized methods established in our clinical laboratory. Furthermore, based on read-length distribution, the proportion of reads shorter than the expected 16S rDNA amplicon length appeared similar across sequencing batches from healthy and immunocompromised BAL specimens (S2 Fig).

The relative abundance of taxa in BAL specimens from immunocompromised patients also diverged markedly from that of healthy controls at all taxonomic levels, showing high inter-sample heterogeneity and a predominance of opportunistic and potentially clinically relevant pathogens over Gram-negative anaerobes (Fig 3). While data remain limited, a prior study compared the BAL microbiota of immunocompromised adults without disease to those with invasive pulmonary aspergillosis [20]. Notably, the microbial composition of BAL specimens from immunocompromised patients in our study contrasts with that of immunocompromised adults without disease, whose microbiota resembled that of our healthy control cohort. These findings suggest that lower respiratory tract infections or their treatment may significantly alter the microbial landscape in immunocompromised individuals.

An assessment of bacterial species diversity within specimens, based on various alpha diversity indices, revealed that both species richness and evenness were significantly higher in the healthy control cohort compared to the immunocompromised cohort, suggesting a potential effect of broad antimicrobial usage in the immunocompromised group. On the other hand, both the PCoA plot based on the Bray–Curtis and Jaccard dissimilarity matrices demonstrated that the bacterial communities in BAL specimens from the immunocompromised cohort were highly heterogeneous, showing greater variability compared to the more tightly clustered specimens from the healthy control cohort (Fig 2B).

To assess the potential diagnostic performance of 16S rRNA gene sequencing for detecting bacterial pathogens in BAL specimens from immunocompromised patients with pneumonia, we identified species that were differentially abundant in the BAL of immunocompromised patients compared to the healthy cohort using MaAsLin2 — a statistical framework designed to identify associations between microbial features and metadata while accounting for potential confounders. It uses generalized linear models with support for data normalization, transformation, and multiple testing correction, making it well suited for high-dimensional microbiome datasets. [18]. As expected, several Gram-negative anaerobes that were most abundant in healthy BAL showed a negative log fold change (LFC) in the immunocompromised cohort (Fig 5, S3 Table). In contrast, several potentially clinically significant taxa showed a positive LFC in the immunocompromised cohort. These include opportunistic atypical pathogens such as *Mycoplasma* species (excluding *M. pneumoniae*) and *Nocardia* species, which are often missed by routine culture and PCR-based diagnostic methods. Other species with a positive LFC include *Pseudomonas aeruginosa*, a well-recognized respiratory pathogen that may have been missed by culture due to prior antibiotic treatment. The clinical significance of lactic acid bacteria (e.g., *Lacticaseibacillus rhamnosus*) —generally considered beneficial—also warrants careful evaluation, as infections caused by these bacteria can occur in immunocompromised hosts. Similarly, the roles of *S. intermedius* and *S. epidermidis*, both of which showed high LFCs, need to be carefully assessed due to the possibility of contamination during sample collection and processing. While our differential abundance analysis revealed several potentially clinically significant taxa, further studies are needed to establish a causal link between these organisms and pneumonia in immunocompromised hosts.

This study has several limitations that should be considered when interpreting the findings. The overall sample size was limited, which may have reduced the statistical power to detect subgroup-specific differences. Notably, there was also a demographic imbalance between the study cohorts, particularly with respect to age, which may act as a confounding factor influencing microbiome composition independent of disease status. In addition, although efforts were made to capture antimicrobial exposure through retrospective chart review, antibiotic history was incomplete for some patients, and prior antimicrobial use, especially in the immunocompromised cohort, may have significantly impacted microbial community structure. These factors limit the ability to attribute observed differences solely to disease state. Accordingly, the comparative conclusions should be interpreted with caution, and future studies incorporating better-matched cohorts and more comprehensive clinical metadata, along with appropriate statistical adjustment for confounders, are warranted.

Furthermore, potential batch effects related to differences in sample collection sources and time periods may have influenced the results. Although all specimens were stored under identical conditions and processed using the same standardized laboratory protocols, differences in collection context and temporal distribution cannot be fully excluded as sources of variability.

In conclusion, 16S rRNA gene-based microbiome analysis of BAL specimens from immunocompromised patients with pneumonia revealed significant dysbiosis and an imbalance in the lower respiratory microbiota compared to healthy individuals. Additionally, since most specimens analyzed in this study were culture-negative or showed insignificant growth, our findings highlight the potential diagnostic utility of the 16S metagenomic approach in detecting atypical and opportunistic bacterial pathogens that are often missed by routine culture. The homogeneity of taxa and close clustering observed in the healthy BAL microbiota in our study further supports the use of 16S metagenomics as a potential diagnostic tool for pneumonia in immunocompromised patients. Common taxa identified in the healthy cohort can serve as a background model to aid accurate interpretation of 16S metagenomic results in immunocompromised populations. Further studies are needed to evaluate the diagnostic performance and clinical impact of using 16S metagenomics for bacterial pathogen detection in BAL specimens from immunocompromised patients.

## Supporting information

S1 Table Standard culture and PCR results of bronchoalveolar lavage (BAL) specimens from immunocompromised patients. (PDF)

S2 Table Verification of 16S rRNA gene amplification and sequencing workflows using cultured bacterial isolates as reference standards. (PDF)

S3 Table Differentially abundant species (q < 0.1) in the immunocompromised cohort compared to the healthy cohort, identified by MaAsLin2. (PDF)

S4 Table BioProject PRJNA1284946 sample metadata and accession numbers. (PDF)

S1 Fig Relative abundance of bacterial species detected by 16S rRNA gene sequencing of cultured bacterial isolates. (PDF)

S2 Fig Read-length distribution of sequences generated by Nanopore-based 16s rRNA gene sequencing of bronchoalveolar lavage specimens from healthy volunteers and immunocompromised patients. (PDF)
