# Supplementary material for: Microbiome analysis of bronchoalveolar lavage (BAL) specimens from immunocompromised patients with pneumonia compared to those from healthy volunteers
Source: PLoS One. 2026 Jun 10;21(6):e0351562. doi: 10.1371/journal.pone.0351562 (PMC13252719; doi:10.1371/journal.pone.0351562)
Supplement: S1 Table — (PDF) [file pone.0351562.s001.pdf]

S1 Table: Standard culture and PCR results of bronchoalveolar lavage (BAL) specimens from immunocompromised patients.

Sample no	Bacterial culture	Fungal culture	*PCR
IC01	IG	NG	Neg
IC02	IG	NG	Neg
IC03	IG	NFE/NG	Neg
IC04	NG	NFE/NG	Neg
IC05	IG	NFE/Scant Growth of *Aspergillus versicolor* species complex	HSV+ve, CMV+ve
IC06	NG	ND	COVID19+ve
IC7	NG	NFE/1 colony of *Aspergillus fumigatus* species complex	COVID19+ve
IC8	NG	NFE/*Rhizomucor pusillus*	COVID19+ve
IC9	NG	NFE/NG	Neg
IC10	NG	ND	COVID19+ve
IC11	NG	ND	COVID19+ve
IC12	NG	NG	Neg
IC13	NG	ND	COVID19+ve
IC14	NG	NFE/NG	Neg
IC15	NG	NG	Neg
IC16	IG	NFE	Neg
IC17	IG	ND	Neg
IC18	NG	NFE	Neg
IC19	IG	NFE/NG	Neg
IC20	IG	NFE/NG	PJ+ve
IC21	NG	NFE/NG	HSV1+ve, PJ+ve
IC22	NG	NFE/NG	CMV+ve, PJ+ve
IC23	NG	NFE/NG	FluA+ve
IC24	NG	NFE/NG	PJ+ve
IC25	>10^6 CFU/L *Stenotrophomonas maltophilia*	NFE/NG	PJ+ve
IC26	NG	NFE/NG	PJ+ve
IC27	NG	NFE/NG	PJ+ve
IC28	NG	NFE/NG	Neg
IC29	IG	NFE/NG	PJ+ve
IC30	NG	NFE/NG	PJ+ve

IC31	NG	NFE/1 colony of *Penicillium* species	PJ+ve
IC32	NG	NFE/NG	CMV+ve, PJ+ve
IC33	NG	NFE/NG	Neg
IC34	IG	NFE/NG	PJ+ve
IC35	NG	$>10^6$ CFU/L *Candida albicans*	PJ+ve
IC36	IG	NFE/NG	Neg
IC37	IG	NFE/NG	Neg
IC38	IG	NFE/NG	Neg
IC39	$>10^6$ CFU/L *Staphylococcus aureus* MRSA	NFE/NG	Neg
IC40	NG	NFE/NG	FluA+ve
IC41	NG	NFE/NG	Neg
IC42	$>10^6$ CFU/L *Staphylococcus aureus* MRSA	NFE/NG	Neg
IC43	IG	NFE/NG	Neg
IC44	NG	NFE/NG	Neg
IC45	NG	NFE/NG	Neg
IC46	NG	NFE/NG	Neg
IC47	NG	NFE/NG	Neg
IC48	IG	NFE/NG	CMV+ve
IC49	$>10^6$ CFU/L *Staphylococcus aureus*	NFE/NG	Neg
IC50	IG	NFE/NG	HSV1+ve, PJ+ve
IC51	NG	NFE/NG	Neg
IC52	NG	NFE/NG	Neg

*PCR assays include cytomegalovirus (CMV), herpes simplex virus 1 & 2 (HSV1/2), varicella zoster virus (VZV), *Mycoplasma pneumoniae* (MPN), *Chlamydophila pneumoniae* (CPN), Respiratory virus PCR panel, Pneumocystis jiroveci (PJ) and *Legionella pneumophila* (LP)

**IG, low colony count and/or insignificant growth of aerobic non-pathogenic organisms; NG, no growth; NFE, no fungal elements seen by KOH-CALCOFLUOR preparation and microscopy; ND, not done
