# Supplementary material for: Microbiome analysis of bronchoalveolar lavage (BAL) specimens from immunocompromised patients with pneumonia compared to those from healthy volunteers
Source: PLoS One. 2026 Jun 10;21(6):e0351562. doi: 10.1371/journal.pone.0351562 (PMC13252719; doi:10.1371/journal.pone.0351562)
Supplement: S2 Table — (PDF) [file pone.0351562.s002.pdf]

S2 Table: Verification of 16S rRNA gene amplification and sequencing workflows using cultured bacterial isolates as reference standards*

**Organism**	**Strain**	****Species identified by 16s rRNA gene sequencing**	**No of reads aligned to reference sequence (% of total non-host reads)**
Clostridium perfringens	ATCC13124	*Clostridium perfringens*	42,386 (99.8)
Escherichia coli	ATCC13351	*Escherichia fergusonii*	28,326 (47.9)
Escherichia coli	QC strain QMPLS0902-1	*Escherichia marmotae*	44,436 (61.9)
Haemophilus influenzae	ATCC1021	*Haemophilus influenzae Biogroup aegyptius*	31,802 (78.1)
Klebsiella pneumoniae	ATCC700603	*Klebsiella pneumoniae*	38,511 (57.1)
Klebsiella pneumoniae	ATCC BAA-1705	*Klebsiella pneumoniae*	52,183 (73.1)
Neisseria gonorrhoeae	ATCC43069	*Neisseria gonorrhoeae*	47,990 (99.4)
Staphylococcus aureus	ATCC25923	*Staphylococcus aureus*	38,745 (79.7)
Enterobacter cloacae	Clinical isolate	*Enterobacter cloacae*	41,308 (99.7)
Citrobacter freundii	Clinical isolate	*Citrobacter freundii*	24,587 (55.5)
Enterococcus avium	Clinical isolate	*Enterococcus avium*	43,445 (89.6)
Enterococcus faecium	Clinical isolate	*Enterococcus faecalis*	52,029 (99.8)
Streptococcus pneumoniae	ATCC49619	*Streptococcus pneumoniae*	11,556 (94.5)
Staphylococcus aureus	Clinical isolate	*Staphylococcus aureus*	14,258 (79.1)
Mycobacterium fortuitum	Clinical isolate	*Mycolicibacterium fortuitum*	26,818 (99.0)

* Bacterial isolates were collected from the Microbiology Laboratory of the Hamilton Regional Laboratory Medicine Program (HRLMP). For each isolate, a single colony was resuspended in 1 mL of TE buffer (pH 8.0) and heat-treated at 95 °C for 10 minutes. Five microliters of the heat extract was then subjected to 16S rRNA gene amplification and sequencing as described in the Materials and Methods section.

** Only the most abundant species, based on the number of sequence reads aligning to the reference sequence, is listed in the table.
