# Supplementary material for: Microbiome analysis of bronchoalveolar lavage (BAL) specimens from immunocompromised patients with pneumonia compared to those from healthy volunteers
Source: PLoS One. 2026 Jun 10;21(6):e0351562. doi: 10.1371/journal.pone.0351562 (PMC13252719; doi:10.1371/journal.pone.0351562)
Supplement: S3 Table — (PDF) [file pone.0351562.s003.pdf]

S3 Table: Differentially abundant species (q < 0.1) in the immunocompromised cohort compared to the healthy cohort, identified by MaAsLin2

Feature	coef	stderr	N	N.not.0	pval	qval
Prevotella jejuni	-8.5002238	0.70223827	72	27	7.87E-19	9.99E-17
Fusobacterium nucleatum	-6.8165799	0.57443519	72	27	2.02E-18	1.28E-16
Selenomonas sputigena	-5.5569198	0.51540797	72	22	1.59E-16	6.74E-15
Fusobacterium pseudoperiodonticum	-6.4442139	0.61784695	72	29	6.74E-16	2.14E-14
Capnocytophaga leadbetteri	-4.6432288	0.45794446	72	18	2.24E-15	5.69E-14
Peptostreptococcus stomatis	-4.7489057	0.47505306	72	17	4.05E-15	7.45E-14
Prevotella vespertina	-6.6476966	0.66520002	72	27	4.11E-15	7.45E-14
Prevotella pallens	-10.373676	1.05000628	72	32	6.59E-15	1.05E-13
Fusobacterium periodonticum	-7.0070521	0.71599165	72	26	9.72E-15	1.37E-13
Prevotella histicola	-8.3707391	0.87102035	72	34	2.03E-14	2.58E-13
Oribacterium asaccharolyticum	-2.977805	0.3498607	72	23	2.09E-12	2.41E-11
Streptococcus symci	-4.9301358	0.60260489	72	24	8.46E-12	8.96E-11
Leptotrichia wadei	-5.1337267	0.64574388	72	24	2.26E-11	2.21E-10
Selenomonas infelix	-5.171288	0.65238843	72	18	2.49E-11	2.26E-10
Catonella massiliensis	-3.2905566	0.4188558	72	15	3.36E-11	2.85E-10
Selenomonas felix	-8.3555995	1.07629782	72	44	4.98E-11	3.96E-10
Treponema medium	-2.7788239	0.40658604	72	12	2.50E-09	1.87E-08
Streptococcus toyakuensis	-3.536346	0.52782003	72	23	4.38E-09	3.09E-08
Campylobacter showae	-2.9046076	0.43928436	72	15	6.31E-09	4.22E-08
Campylobacter massiliensis	-2.4562829	0.38353943	72	16	1.49E-08	9.49E-08
Streptococcus oralis	-4.7373209	0.7522583	72	27	2.32E-08	1.34E-07
Prevotella salivae	-5.7756775	0.91557791	72	42	2.22E-08	1.34E-07
Veillonella atypica	-7.9448723	1.26486985	72	43	2.48E-08	1.37E-07
Streptococcus vulneris	-3.1212123	0.50114171	72	20	3.08E-08	1.63E-07
Streptococcus downii	-3.1063793	0.50957504	72	18	5.30E-08	2.69E-07
Neisseria perflava	-6.5221629	1.0773113	72	33	6.29E-08	3.07E-07
Selenomonas flueggei	-2.6936492	0.44730858	72	13	7.17E-08	3.37E-07
Streptococcus mitis	-3.0065532	0.51295516	72	28	1.37E-07	6.23E-07
Oribacterium parvum	-2.6436915	0.46626027	72	15	2.96E-07	1.30E-06
Campylobacter concisus	-4.029177	0.71397784	72	38	3.30E-07	1.40E-06
Streptococcus intermedius	6.0961143	1.08287365	72	39	3.48E-07	1.43E-06
Prevotella melaninogenica	-6.353779	1.14303742	72	47	4.62E-07	1.83E-06
Capnocytophaga granulosa	-3.4248899	0.62081881	72	13	5.45E-07	2.10E-06
Haemophilus parainfluenzae	-4.9632443	0.90280647	72	30	5.88E-07	2.20E-06
Granulicatella adiacens	-3.7032225	0.68721389	72	22	9.04E-07	3.28E-06

Capnocytophaga gingivalis	-2.799931	0.53134017	72	15	1.44E-06	5.08E-06
Eubacterium sulci	-1.5113525	0.28952329	72	10	1.75E-06	5.87E-06
Prevotella shahii	-3.1086911	0.59568913	72	12	1.76E-06	5.87E-06
Veillonella tobetsuensis	-3.5940908	0.69723118	72	22	2.25E-06	7.32E-06
Oribacterium sinus	-2.3134645	0.45188426	72	19	2.58E-06	8.18E-06
Streptococcus pseudopneumoniae	-2.5113283	0.49384817	72	16	2.94E-06	9.11E-06
Campylobacter rectus	-2.3361622	0.46652743	72	12	3.96E-06	1.17E-05
Prevotella veroralis	-2.4254115	0.48438722	72	17	3.97E-06	1.17E-05
Streptococcus infantis	-3.2078462	0.65265295	72	19	5.63E-06	1.63E-05
Fusobacterium canifelinum	-1.6035406	0.33221245	72	11	7.86E-06	2.22E-05
Candidatus Peptostreptococcus massiliensis	-1.8059633	0.37622182	72	11	8.68E-06	2.40E-05
Streptococcus salivarius	-4.0867457	0.87570125	72	23	1.43E-05	3.86E-05
Lachnoanaerobaculum gingivalis	-1.6062717	0.34487717	72	13	1.48E-05	3.91E-05
Porphyromonas pasteri	-3.6238719	0.78401686	72	30	1.69E-05	4.37E-05
Alloprevotella rava	-2.4308854	0.52665151	72	13	1.73E-05	4.38E-05
Enterococcus gallinarum	5.1037727 4	1.1117237	72	33	1.89E-05	4.71E-05
Fusobacterium watanabei	-2.312306	0.51405384	72	10	2.66E-05	6.49E-05
Stenotrophomonas maltophilia	4.4565209 1	1.01139784	72	33	3.71E-05	8.88E-05
Aggregatibacter aphrophilus	-1.8582985	0.43111884	72	12	5.23E-05	0.00012073
Fusobacterium hwasookii	-2.3213599	0.53819282	72	14	5.18E-05	0.00012073
Escherichia fergusonii	3.9798274 7	0.95826708	72	31	9.12E-05	0.0002068
Prevotella oris	-3.7591214	0.91437876	72	33	0.00010561	0.00023531
Streptococcus gwangjuense	-2.4253398	0.59292905	72	17	0.00011349	0.00024851
Veillonella rogosae	-3.7558353	0.92202013	72	30	0.00012038	0.00025911
Capnocytophaga ochracea	-1.6087362	0.39829702	72	9	0.00013562	0.00028706
Megasphaera micronuciformis	-2.596818	0.67031697	72	26	0.00023827	0.00049121
Veillonella nakazawae	-3.7918208	0.97926263	72	34	0.0002398	0.00049121
Solobacterium moorei	-1.8316201	0.47626416	72	18	0.00026203	0.00052822
Capnocytophaga sputigena	-2.2973043	0.60090314	72	15	0.0002828	0.00056119
Shigella flexneri	4.1552965 4	1.09514268	72	27	0.00031139	0.00060842
Prevotella nanceiensis	-3.8225104	1.00967897	72	23	0.00032028	0.00061629
Selenomonas timonae	-1.8594486	0.49988958	72	9	0.00039887	0.00075608
Alloprevotella tannerae	-2.1248584	0.572708	72	11	0.00041161	0.00076873
Porphyromonas endodontalis	-3.4142972	0.95119547	72	30	0.00061053	0.00112372
Campylobacter gracilis	-2.4086172	0.67190857	72	18	0.00061996	0.00112478
Selenomonas noxia	-2.5437291	0.7653316	72	16	0.0014157	0.00253231

Neisseria macacae	-1.3343184	0.41296505	72	9	0.00188065	0.00331726
Parvimonas micra	-1.4004402	0.43928737	72	11	0.00214269	0.00372769
Streptococcus shenyangsis	-1.6267305	0.52053767	72	12	0.00258704	0.00443992
Lautropia mirabilis	-0.8358556	0.26886343	72	9	0.00271511	0.00459758
Staphylococcus epidermidis	2.79037979	0.90109493	72	22	0.00281508	0.00470414
Lacticaseibacillus rhamnosus	2.68048119	0.88332114	72	20	0.0033797	0.0055743
Streptococcus vestibularis	-1.5286888	0.50554464	72	15	0.00348705	0.00567764
Tannerella serpentiformis	-1.8585374	0.62300296	72	13	0.00392435	0.00630877
Gemella sanguinis	-1.175769	0.40882468	72	11	0.00533328	0.00846658
Gemella taiwanensis	-1.8220202	0.64173585	72	15	0.00591523	0.0092745
Catonella morbi	-0.840717	0.30124258	72	9	0.00677	0.01048524
Streptococcus timonensis	-1.4235283	0.51888884	72	11	0.00771645	0.0118071
Haemophilus sputorum	-1.149318	0.42036668	72	8	0.00791644	0.0119689
Prevotella oulorum	-1.8017415	0.66089437	72	19	0.00808871	0.01208548
Gemella haemolysans	-1.6326908	0.59992523	72	15	0.00819384	0.01210021
Haemophilus haemolyticus	-1.9903202	0.73458115	72	11	0.00846705	0.01235994
Lachnoanaerobaculum orale	-1.8950845	0.71494836	72	20	0.00992633	0.0143255
Pseudomonas aeruginosa	2.29885112	0.87707696	72	17	0.01074504	0.0153328
Stomatobaculum longum	-1.0793313	0.42943932	72	21	0.01426351	0.02012739
Gemella morbillorum	-1.1529873	0.49798569	72	9	0.02353278	0.03284245
Escherichia coli	1.64456005	0.73110001	72	14	0.02763234	0.03814464
Pantoea septica	2.04953508	0.92164663	72	13	0.02939209	0.04013759
Streptococcus rubneri	-1.0563975	0.47801752	72	16	0.03038018	0.04104556
Nocardia wallacei	1.83338815	0.85358194	72	13	0.03518682	0.04703922
Prevotella scopos	-0.863622	0.40623984	72	12	0.03704142	0.04900271
Streptococcus thermophilus	2.08959429	0.99685324	72	19	0.03968173	0.05133271
Staphylococcus roterodami	-2.6730297	1.27739601	72	37	0.04001526	0.05133271
Streptococcus parasanguinis	-2.7580813	1.31762238	72	35	0.03995402	0.05133271
Veillonella dispar	-2.8632636	1.40099655	72	39	0.04474451	0.05682553
Staphylococcus capitis	1.35109815	0.66322076	72	11	0.04541485	0.0571058
Bacteroides heparinolyticus	1.17904965	0.60824258	72	10	0.0566011	0.07047392
Lancefieldella parvula	-1.1973849	0.62395052	72	26	0.05905709	0.07281797
Metamycoplasma salivarium	2.03882723	1.1416003	72	38	0.07844029	0.09578766
