# Supplementary material for: Microbiome analysis of bronchoalveolar lavage (BAL) specimens from immunocompromised patients with pneumonia compared to those from healthy volunteers
Source: PLoS One. 2026 Jun 10;21(6):e0351562. doi: 10.1371/journal.pone.0351562 (PMC13252719; doi:10.1371/journal.pone.0351562)
Supplement: S4 Table — (PDF) [file pone.0351562.s004.pdf]

S4 Table: BioProject PRJNA1284946 sample metadata and accession numbers

Group	Sample ID	Accession
Healthy cohort	HC01	SRR34340512
	HC02	SRR34340511
	HC03	SRR34340510
	HC04	SRR34340509
	HC05	SRR34340508
	HC06	SRR34340506
	HC07	SRR34340505
	HC08	SRR34340504
	HC09	SRR34340503
	HC10	SRR34340502
	HC11	SRR34340501
	HC12	SRR34340500
	HC13	SRR34340499
	HC14	SRR34340498
	HC15	SRR34340497
	HC16	SRR34340495
	HC17	SRR34340494
	HC18	SRR34340493
	HC19	SRR34340492
	HC20	SRR34340491
Immunocompromised cohort	IC01	SRR34340530
	IC02	SRR34340529
	IC03	SRR34340518
	IC04	SRR34340507
	IC05	SRR34340496
	IC06	SRR34340490
	IC07	SRR34340489
	IC08	SRR34340488
	IC09	SRR34340487
	IC10	SRR34340486
	IC11	SRR34340528
	IC12	SRR34340527
	IC13	SRR34340526
	IC14	SRR34340525
	IC15	SRR34340524
	IC16	SRR34340523
	IC17	SRR34340522
	IC18	SRR34340521
	IC19	SRR34340520
	IC20	SRR34340519
	IC21	SRR34340517
	IC22	SRR34340516
	IC23	SRR34340515
	IC24	SRR34340514
	IC25	SRR34340513
