# Supplementary material for: Microbiome analysis of bronchoalveolar lavage (BAL) specimens from immunocompromised patients with pneumonia compared to those from healthy volunteers
Source: PLoS One. 2026 Jun 10;21(6):e0351562. doi: 10.1371/journal.pone.0351562 (PMC13252719; doi:10.1371/journal.pone.0351562)
Supplement: S1 Fig — (PDF) [file pone.0351562.s005.pdf]

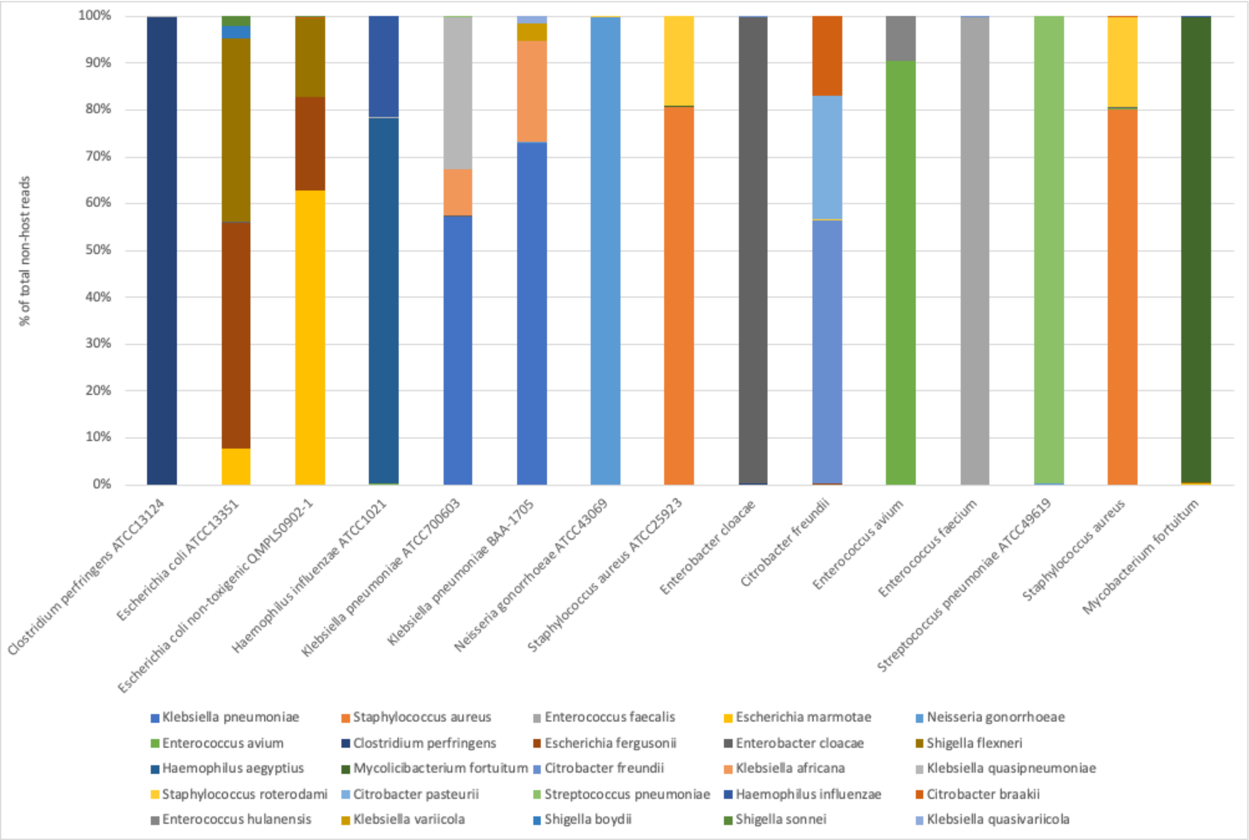

S1 Fig: Relative abundance of bacterial species detected by 16S rRNA gene sequencing of cultured bacterial isolates
