# Supplementary material for: Microbiome analysis of bronchoalveolar lavage (BAL) specimens from immunocompromised patients with pneumonia compared to those from healthy volunteers
Source: PLoS One. 2026 Jun 10;21(6):e0351562. doi: 10.1371/journal.pone.0351562 (PMC13252719; doi:10.1371/journal.pone.0351562)
Supplement: S2 Fig — (PDF) [file pone.0351562.s006.pdf]

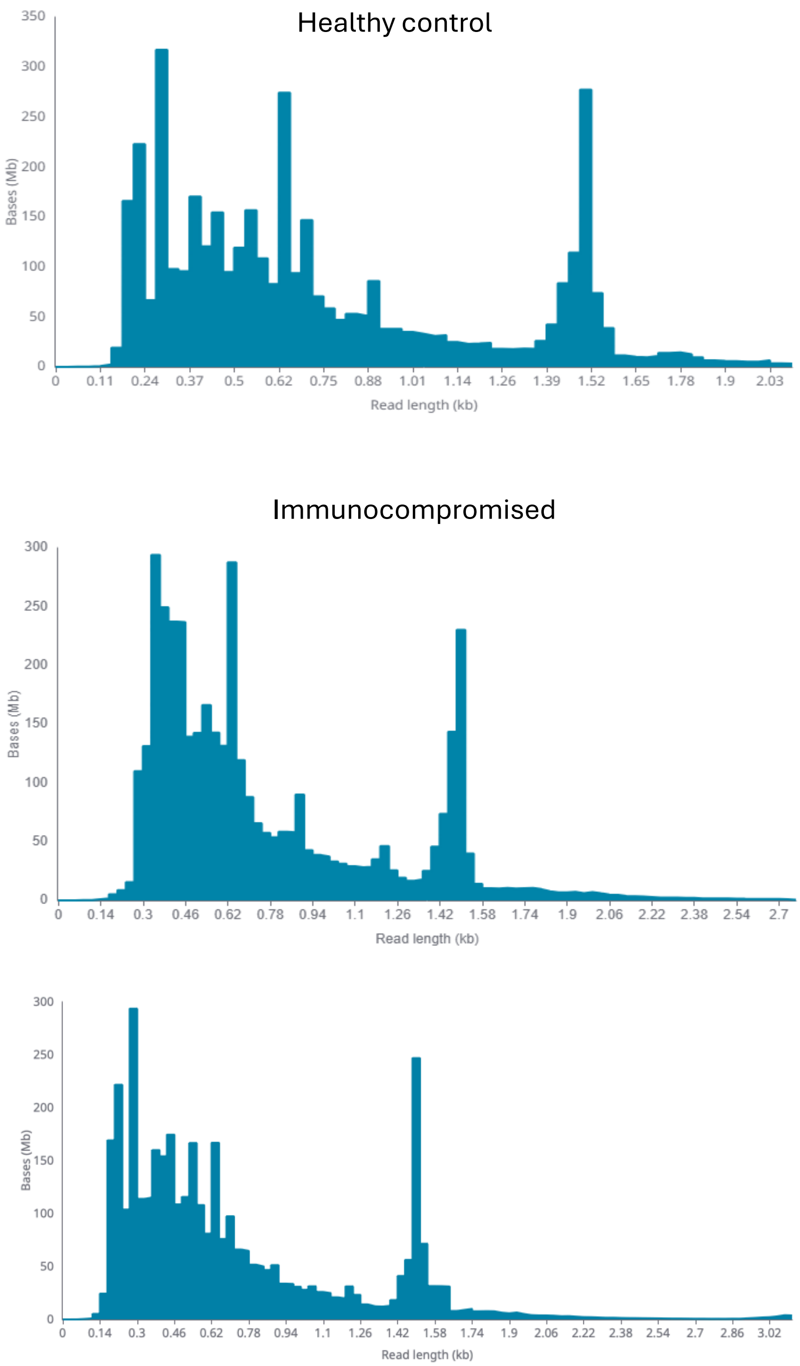

S2 Fig: Read-length distribution of sequences generated by Nanopore-based 16s rRNA gene sequencing of bronchoalveolar lavage specimens from healthy volunteers and immunocompromised patients.
